# Incidental Diagnosis of Healed Aortic Abscess Cavity

**DOI:** 10.7759/cureus.15632

**Published:** 2021-06-14

**Authors:** Shilpa Shree, Vijayanand Palanisamy, Kumar Chidambaram, Vasanthi Vajjiram, Pradeep Ramkoomar

**Affiliations:** 1 Department of Cardiothoracic Surgery, The Madras Medical Mission Hospital, Chennai, IND; 2 Department of Cardiac Anesthesia, The Madras Medical Mission Hospital, Chennai, IND

**Keywords:** abscess, cavity, infective endocarditis, aortic, echocardiography, valve, commissure, mitral leaflet

## Abstract

In infective endocarditis, the perivalvular abscess is a known complication with an incidence of more than 22%-29%, but the primary presentation of a healed aortic abscess without any clinical features of infective endocarditis is very rare. These sorts of cases are scarcely documented throughout literature. We present a successful surgical closure of healed perivalvular abscess cavity with aortic valve replacement and mitral valve repair.

## Introduction

A perivalvular abscess is a known complication of infective endocarditis (IE) with an incidence of around 22%-29% [1,2]. Silent presentation of IE with a healed aortic abscess is very rare, with seldom documented cases throughout literature. Initially, two-dimensional echocardiography has shown to be useful in the diagnosis of abscess cavities. Due to the proximity of transesophageal echocardiography (TEE) probe to the aortic valve (AV) and higher signal-to-noise ratio, TEE was found to be a superior imaging modality in comparison to transthoracic echocardiography (TTE) [3,4]. IE usually presents with symptoms of fever, myalgia, flu-like symptoms, fatigue, chest pain, and signs like heart murmurs, anemia, or embolic events. Our patient presented with only complaints of breathlessness without any other clinical evidence of IE. Intraoperative TEE accidentally picked a rare healed aortic abscess cavity with a floor formed by the left atrium. We report a successfully performed direct closure of a healed perivalvular abscess cavity by including the cavity during AV implantation within the suture line itself.

## Case presentation

**Figure 1:**
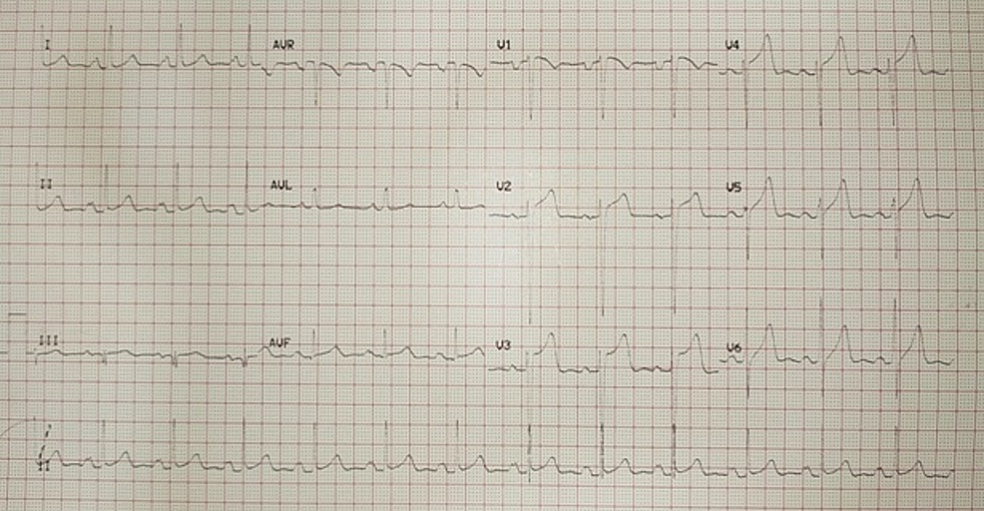
Electrocardiography showing left ventricular hypertrophy and left atrial enlargement

A 44-year-old gentleman presented with complaints of shortness of breath of 30 days' duration. On initial evaluation, his heart rate was 72 beats/min, blood pressure was 120/40 mmHg and he was afebrile. We found an early diastolic murmur in the aortic area and a pansystolic murmur in the mitral area. Electrocardiography (ECG) was suggestive of left ventricular (LV) hypertrophy and left atrial enlargement (Figure 1).

**Figure 2:**
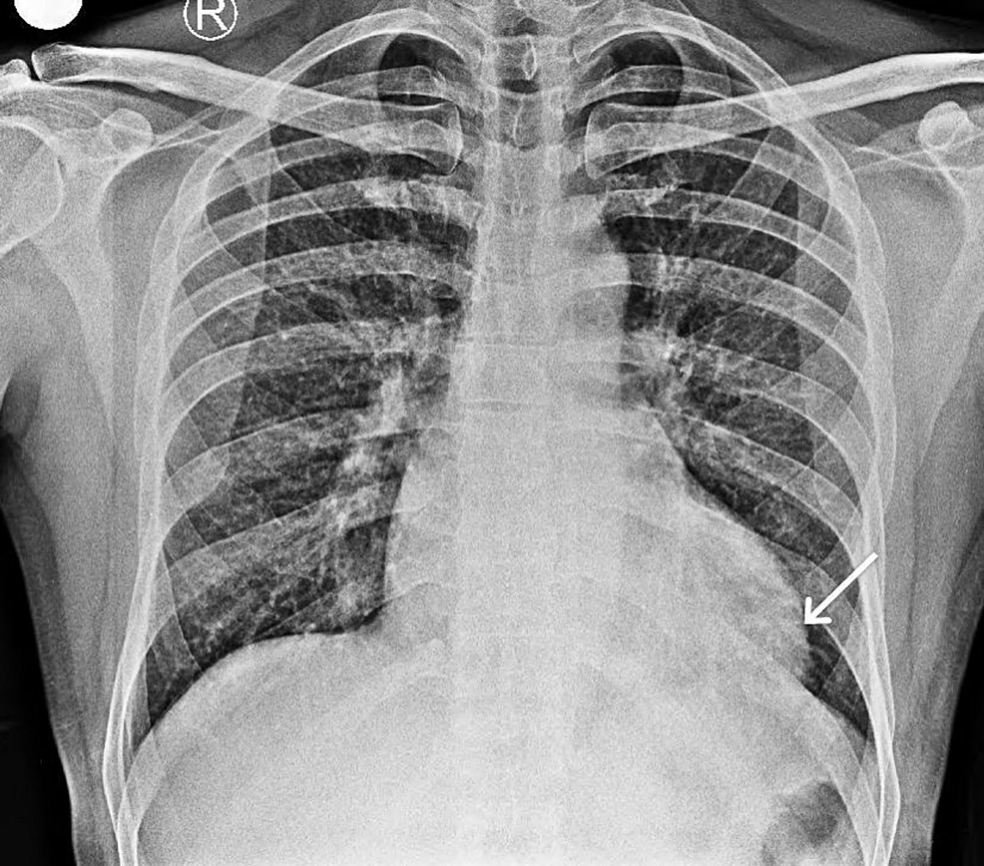
Chest roentgenogram Arrow pointing left ventricular enlargement.

Chest roentgenogram showed LV enlargement (Figure 2).

**Figure 3:**
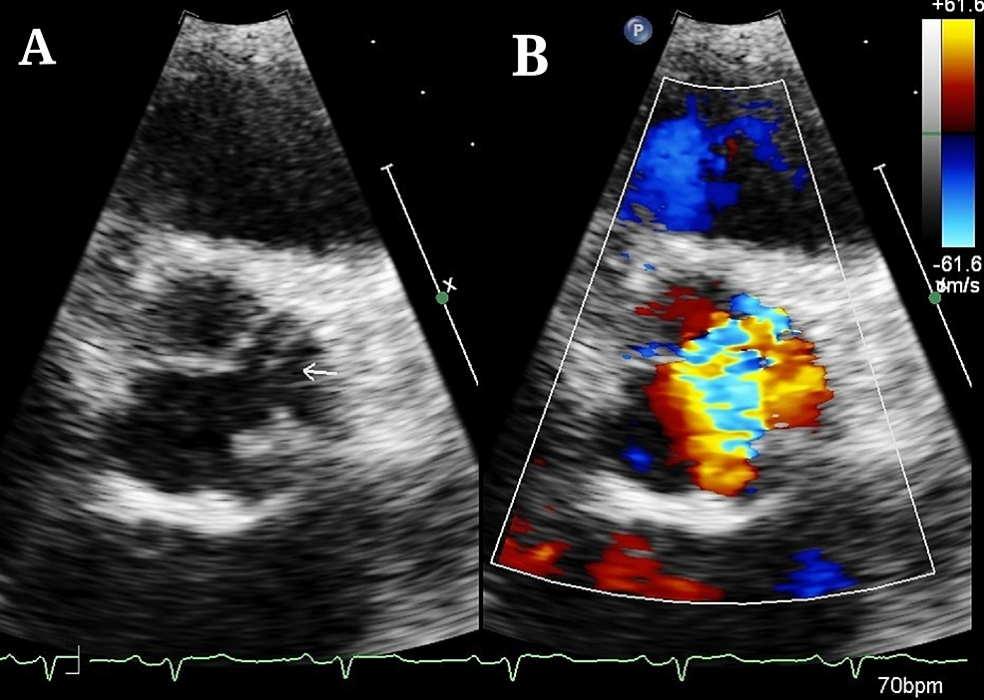
Transthoracic echocardiography - short-axis view (A) Plain image with arrow showing left coronary cusp prolapse. (B) Color Doppler image showing regurgitation jet.

**Figure 4:**
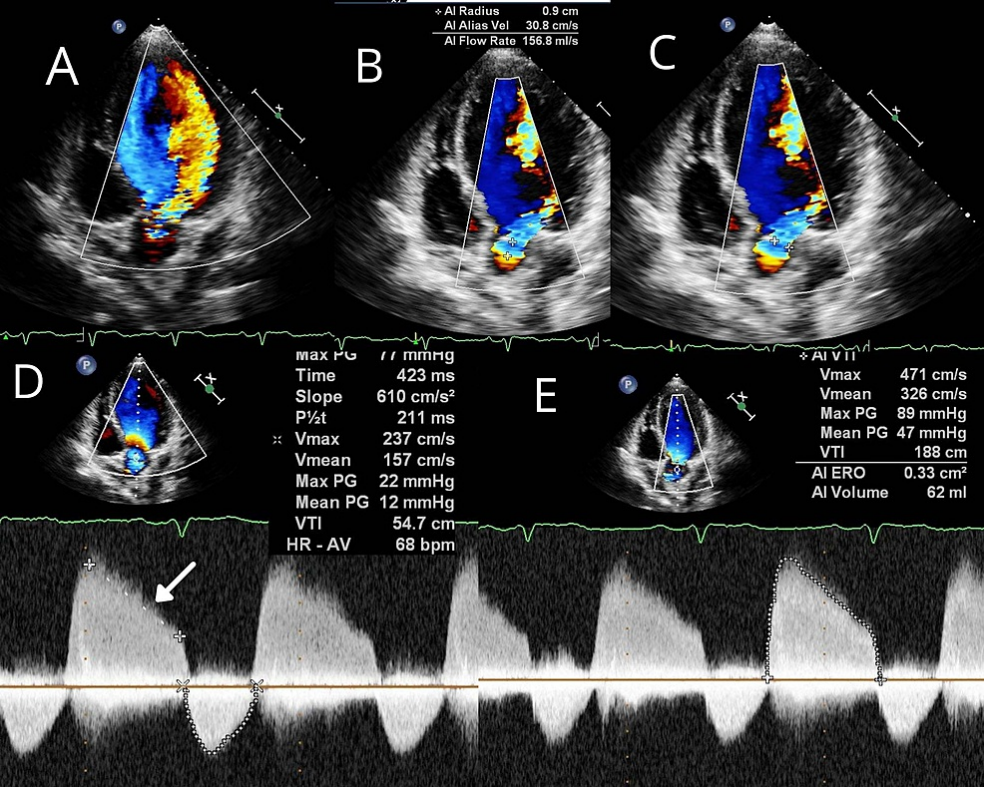
Transthoracic echocardiogram of aortic regurgitation (A) Five-chamber view showing eccentric aortic regurgitation. (B) Five-chamber view showing proximal isovelocity surface area of 0.9 cm. (C) Five-chamber view showing vena contracta 0.883 cm. (D) Five-chamber view with continuous-wave Doppler showing aortic deceleration slope 610 cm/s^2 ^and pressure half time 211 ms. (E) Five-chamber view with continuous-wave Doppler showing effective regurgitant orifice area of 0.33 cm^2^.

**Figure 5:**
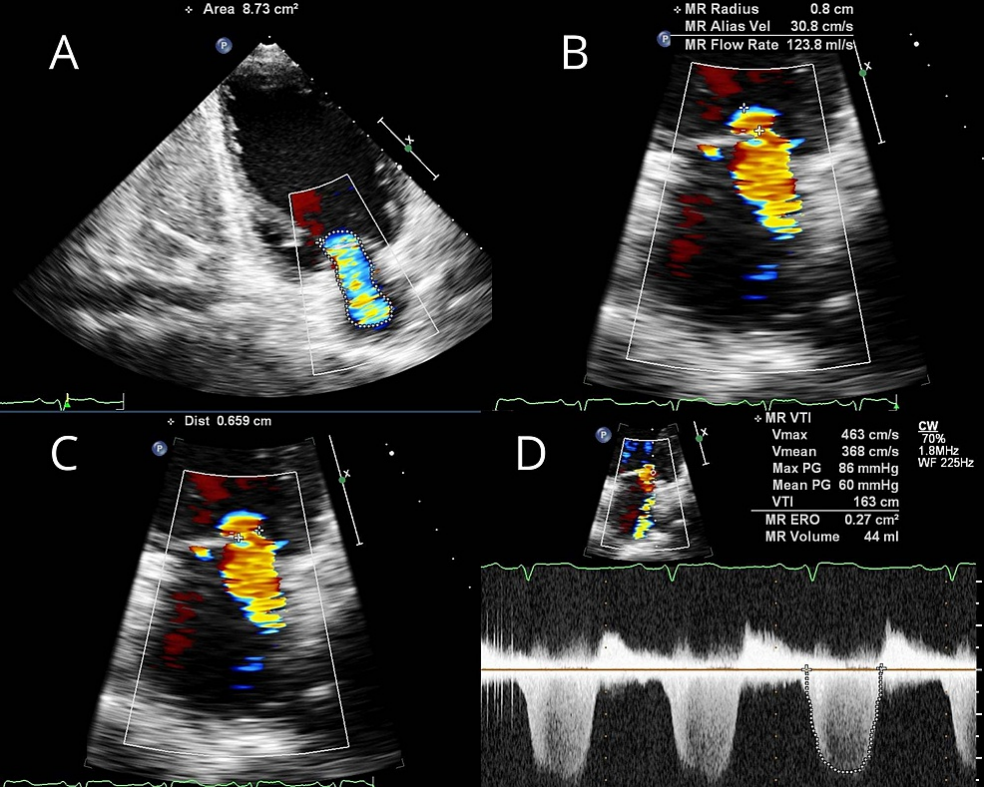
Transthoracic echocardiogram of mitral regurgitation (A) Two-chamber view showing mitral regurgitation with jet area 8.73 cm^2^. (B) Four-chamber view showing proximal isovelocity surface area of 0.8 cm. (C) Four-chamber view showing vena contracta of 0.659 cm. (D) Four-chamber view with continuous-wave Doppler showing effective regurgitant orifice area of 0.27 cm^2^.

TTE (Figures 3A, 3B, 4A-4E, and 5A-5D) revealed severe eccentric aortic regurgitation (AR) (slope - 610 cm/s^2^) with thickened and non-coapting AV, left coronary cusp (LCC) prolapsed, and moderate mitral regurgitation (MR) (jet area - 8.7 sqcm), moderate pulmonary hypertension, dilated LV - left ventricle internal diameter (LVID) diastolic - 69 mm, and LVID (systolic) - 56 mm, and moderate LV systolic dysfunction (ejection fraction - 38%). Other valves were normal. The aortic annulus measured 2.2 cm, ascending aorta 3.2 cm, and sinotubular junction 3.1 cm. He was scheduled to undergo AV replacement with the mitral repair.

No history and clinical features suggestive of IE were found. Hematological and biochemical markers of endocarditis like total leucocyte count (7,300 cells/mm^3^) and erythrocyte sedimentation rate (8 mm/hr) were within the normal range.

**Figure 6:**
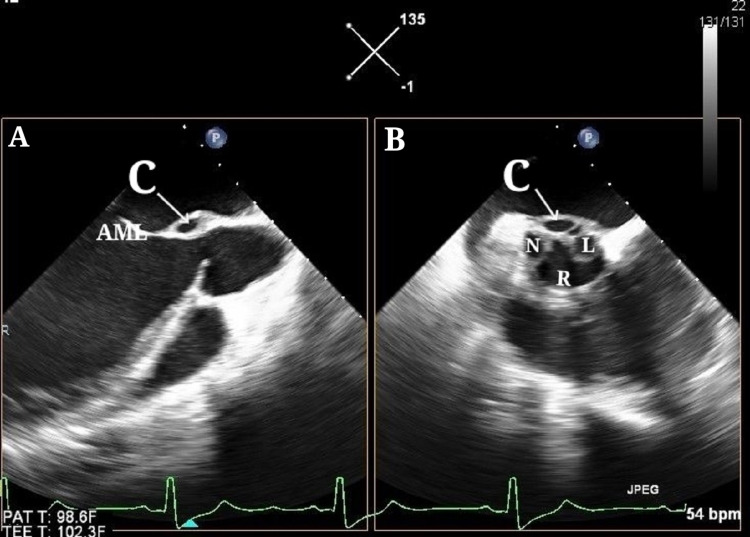
Transesophageal echocardiogram (A) Orthogonal image 90-degree showing the cavity near AML. (B) X-plane or aortic valve short axis showing the cavity at NCC-LCC commissure. C - cavity, N - noncoronary cusp, R - right coronary cusp, L - left coronary cusp, AML - anterior mitral leaflet.

Intraoperative TEE examination showed that the AV is trileaflet with severe AR and showed a cavity (Figures 6A, 6B) near the non-coronary cusp - left coronary cusp (NCC-LCC) commissure extending to the base of anterior mitral leaflet and moderate MR. Three-dimensional (3D) TEE with the multi-beta acquisition with six ECG beats stitched together was done, which confirmed the same findings.

**Figure 7:**
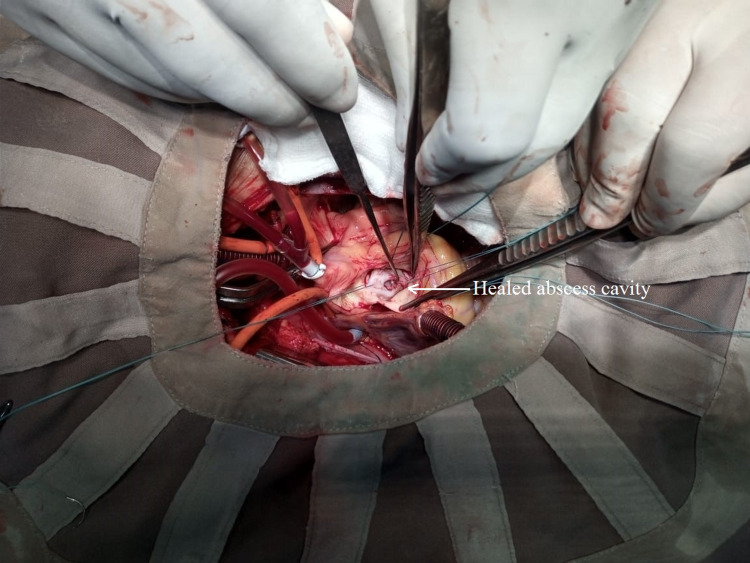
Intra-operative image showing the abscess cavity nearer to NCC-LCC commissure with left atrial musculature at its base

Post sternotomy and pericardiotomy, we found a hugely dilated heart with moderate dilatation of the aorta. Cardiopulmonary bypass (CPB) was instituted using aorto-bicaval cannulation. Hypothermic Delnido cardioplegia was used for diastolic cardiac arrest. After aortotomy, we found a tricuspid AV with thickened and tethered non-coapting cusps. A healed aortic abscess cavity 1 * 1 * 0.5 cm was seen at the NCC sinus close to NCC- LCC commissure (Figure 7).

**Figure 8:**
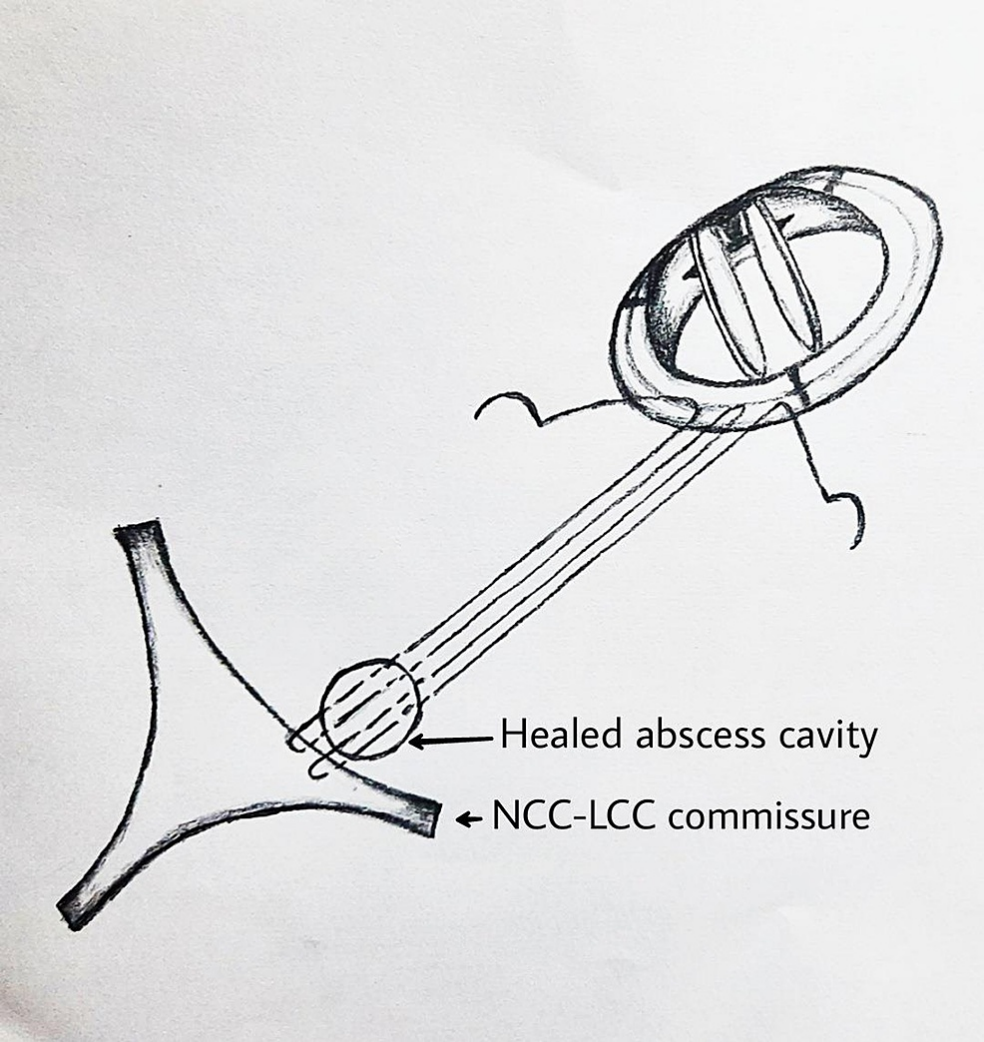
Schematic diagram showing placement of valve implantation sutures along with obliteration of abscess cavity.

Left atrial wall musculature was seen at the base of the cavity. We excised the native AV. A 24-mm aortic mechanical valve was implanted in the aortic position with continuous suture technique. The healed abscess cavity was directly obliterated by including it in the suture line and plicated along with the sewing ring of the prosthetic valve (Figure 8).

**Figure 9:**
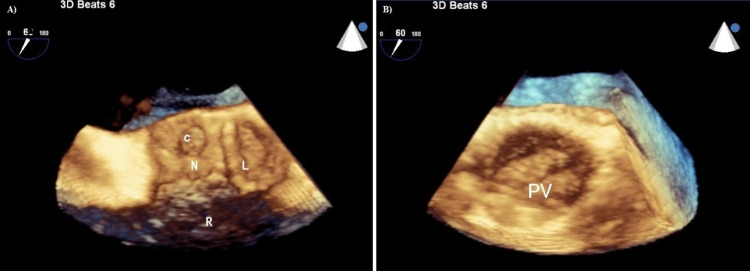
3D zoom crop image obtained by slicing aortic valve deeper (A) Pre-operative image showing the cavity in proximity to NCC, which goes onto the base of AML. (B) Post-operative image showing obliterated cavity with prosthetic valve implantation. C - cavity, N - noncoronary cusp, R - right coronary cusp, L - left coronary cusp, PV - prosthetic valve.

**Figure 10:**
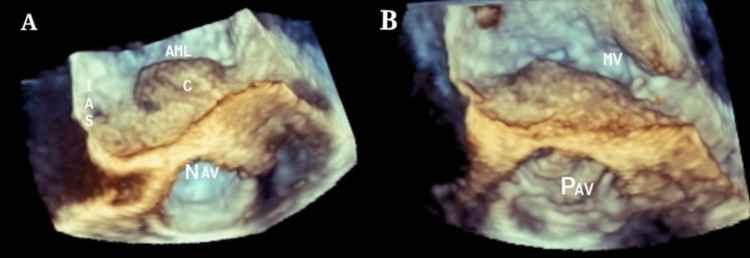
3D zoom image – multi-beta acquisition six ECG beats stitched together (A) Pre-operative image showing cavity extending into base of AML. (B) Post-operative image showing obliterated cavity post aortic valve implantation. IAS - inter-atrial septum, AML - anterior mitral leaflet, C - cavity, NAV - native aortic valve, PAV - prosthetic aortic valve, MV - mitral valve.

**Figure 11:**
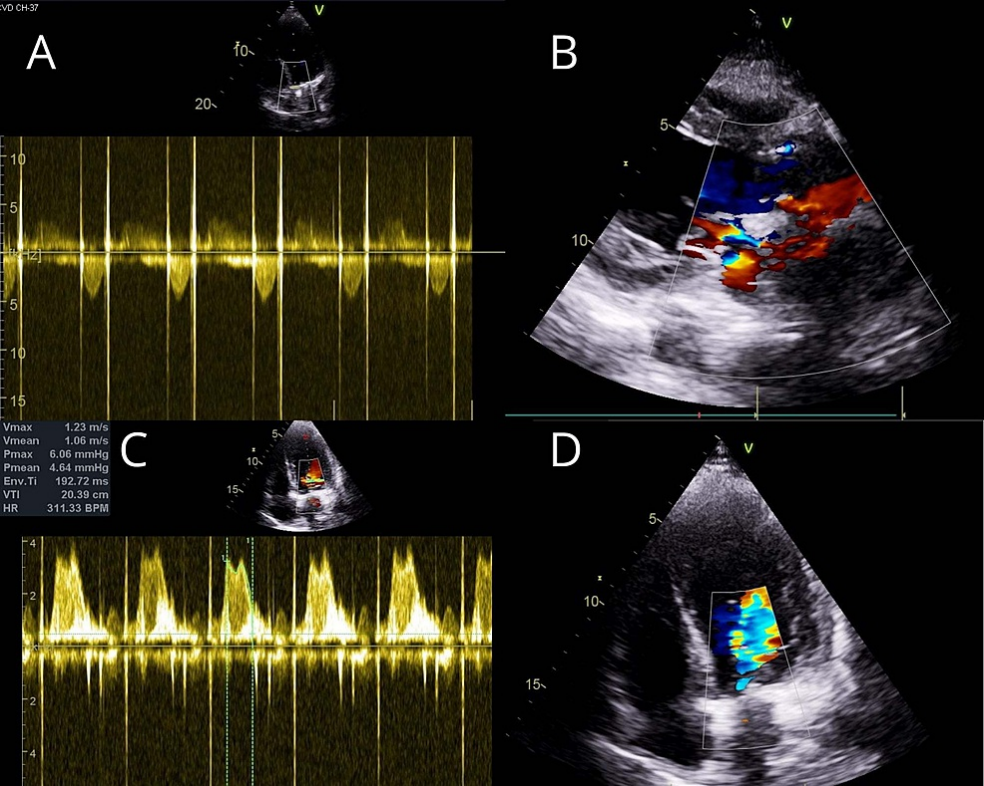
Postoperative transthoracic echocardiography (A) Five-chamber view with continuous-wave Doppler showing no paravalvular leak. (B) Parasternal long-axis view showing no paravalvular leak. (C) Four-chamber view with continuous-wave Doppler showing no residual mitral regurgitation. (D) Four-chamber view showing no mitral regurgitation.

Posterior segmental suture annuloplasty was done in the mitral valve. The patient was weaned off bypass uneventfully with aortic cross-clamp time being 82 minutes and CPB time being 116 minutes. Post-operative TEE had shown no paravalvular leak (Figures 9A, 9B, and 10A, 10B), normally functioning aortic prosthesis and no MR, which was reconfirmed in the seventh post-operative day TTE (Figures 11A-11D).

The patient’s course in the hospital was uneventful. The patient was discharged with antiplatelet, anticoagulant (with target international normalized ratio - 2.5-3.5), and eight weeks of antibiotic course prophylactically (as per our infectious disease physician’s advice).

## Discussion

A paravalvular abscess usually presents as persistent fever, despite appropriate antibiotic therapy, or as a new conduction abnormality as the abscess erodes into adjacent cardiac conduction tissue. Our patient had none of the above symptoms and there was no other history suggestive of IE and presented with a healed abscess cavity, sequelae of IE.

The algorithm proposed by the European Society of Cardiology, The Task Force on the Prevention, Diagnosis, and Treatment of IE recommends TEE in patients with high clinical suspicion of IE along with a normal TTE. TEE is always recommended even when the diagnosis is made, as TTE has the better image quality and sensitivity predominantly for perivalvular involvement. With better quality, negative TTE, and low clinical suspicion of IE, only TTE may be considered satisfactory. Intraoperative TEE is recommended in all patients of IE undergoing surgery [5]. We did not do a pre-operative TEE assessment, as the patient presented devoid of any IE features.

As an institutional policy, we electively do TEE for all patients with valvular abnormalities. 3D TEE with the multi beta acquisition is also done, as it gives a more defined quality of images.

The various procedures involved in the repair of aortic abscess included AV replacement with pericardial patch closure of the cavity, annular reconstruction, Bentall’s procedure, and other aortic root replacement surgeries.

This case had a healed abscess detected incidentally and a similar case was reported once by Datt et al. where they detected on intraoperative TEE where two healed abscesses were noted. The first was at the NCC-LCC commissure and the other on top of the right coronary cusp. The patient had AV (25 mm mechanical valve) and ascending aorta (26 mm Dacron graft) replacement under standard CPB technique [6].

In our patient, the healed abscess cavity was obliterated by including it in the suture line and plicated along with the AV, without using any patching material. It is always good to avoid prosthetic materials, as the chance for reinfection is high. Croon et al., while analyzing the outcome of AV endocarditis with periannular abscess, has found that the mean aortic cross-clamp time was 110±40 minutes and mean bypass time of 157±70 minutes [7]. Our technique has reduced the aortic cross-clamp and bypass time. The pre-requisite to our technique is that cavity should be nearer to the aortic annulus and should not be too deep. If any suspicion of infection there, then the cavity should be left open.

The left atrial musculature was seen on the floor of our abscess cavity. This shows that on the progression of the abscess it would have developed into a ruptured sinus of valsalva (RSOV) into the left atrium. RSOV rupturing into the left atrium is itself an extremely rare presentation.

## Conclusions

Healed aortic paravalvular abscess is a rare complication of IE. Obliterating the abscess, by simply including it in the suture line while implanting the AV prosthesis is an easier and quicker technique for managing healed periannular abscess. Assessing the characteristics of abscesses with careful selection of surgical techniques is the keystone in the management of these patients.
